# Treatment of Post-Partum Hemorrage by Intra-Uterine Compression of the Abdominal Aorta

**Published:** 1890-05

**Authors:** 


### TREATMENT OF POST-PARTUM HEMORRAGE BY INTRA-UTERINE COMPRESSION OF THE ABDOMINAL AORTA.

The methods for checking post-partum hemorrhage may be divided into two classes: 1st, internal hæmostatic processes, in which drugs are used; 2d, external or obstetrical processes. The first class may be represented by ergot and is to be considered inefficient in case of pressing and threatening danger. The external methods may be divided into extra and intra-uterine varieties. The former are generally used, and include cold applications to the abdomen, ventral compression by the bandage, pressure upon the uterus with the hand, abdominal compression of the aorta, and galvanism. The intra-uterine methods include the introduction of ice within the uterine cavity, lemon-juice vinegar, solutions of iodine, perchloride of iron, or water at 40 deg. to 50 deg. C. An intra-uterine tampon has also been advised. All these measures are useful, but may be inefficient, for they presuppose materials, assistants, and apparatus which may not be at hand when the emergency arises. The method which is here advocated as superior to the others is intra-uterine compression of the abdominal aorta. The right hand having been carefully disinfected with sublimate, is to be passed into the uterine cavity, and the aorta, which can readily be found, is to be compressed against the vertebral column. After compression has been continued for only a few seconds the bleeding will cease. This method has been tried successfully a number of times by the author, by Budiger, of Tubingen, who reported twenty cases in which he had used it, his paper appearing ten or more years ago. Boer and Jaquemin have both tried the method and have disapproved it. The objections which have been made are that the hemorrhage at such a time is venous rather than arterial, and also that compression of the abdominal aorta would not influence the uterine and ovarian arteries. In spite of theoretical obiections the method may prove serviceable in an emergency.—A. F. C. in *Annals of Gynæcology and Pædiatry.*

